# Vacuum-Assisted Closure in a Patient With Entero-Atmospheric Fistula: A Case Report

**DOI:** 10.7759/cureus.12777

**Published:** 2021-01-18

**Authors:** Norah M Alsubaie, Abdullah M Albdah, Nouf R Alrushaid, Fahad Al-Abdullatif, Gaida A Aljamili

**Affiliations:** 1 General Surgery, King Khalid University Hospital, Riyadh, SAU; 2 Trauma Surgery, Prince Mohammed Bin Abdulaziz Hospital, Riyadh, SAU; 3 College of Medicine, King Saud University, Riyadh, SAU

**Keywords:** eaf, vacuum assisted closure, vac, surgery, complication, open surgery, entero-atmospheric fistula

## Abstract

The entero-atmospheric fistula (EAF) is a recognized complication of open abdomen surgeries, which causes significant morbidity and mortality. This usually causes long hospitalizations and may require many surgical operations. While different methods of treatment for EAF are used, all different methods share the same goal, which is a proper closure of the fistula and the open abdomen to avoid recurrence and complications. We report a case of a 48-year-old female with a bowel perforation following an attempted open bilateral ovarian cyst drainage with cyst wall biopsy complicated by entero-atmospheric fistula treated by wound closure with vacuum-assisted pressure. In conclusion, the use of vacuum-assisted closure (VAC) to induce spontaneous healing of EAFs can provide a safe acceptable alternative to surgical treatment.

## Introduction

The sudden appearance of an entero-atmospheric fistula (EAF) following abdominal surgery is considered a devastating complication to both the patient and the surgeon. It is often associated with fluid and electrolyte disbalance, nutritional deficiencies, and life-threatening sepsis [1,2]. Its therapy is therefore challenging and linked to elevated morbidity and mortality [2]. The management of distal low-output fistula often have a benign clinical course, as it often closes spontaneously with conservative management. On the other hand, a proximal high-output fistula has a complicated course and well-known to fail conservative management [3,4]. Spontaneous healing is restrained by the absence of viable vascular tissue coverage over the exposed bowel [3]. There have been several attempts to overcome these issues and promote successful management of such fistulas [4-6]. In this paper, we report a case of a 48-year-old female with a bowel perforation following an attempted open bilateral ovarian cyst drainage with cyst wall biopsy complicated by entero-atmospheric fistula treated by wound closure with vacuum assisted pressure.

## Case presentation

**Figure 1:**
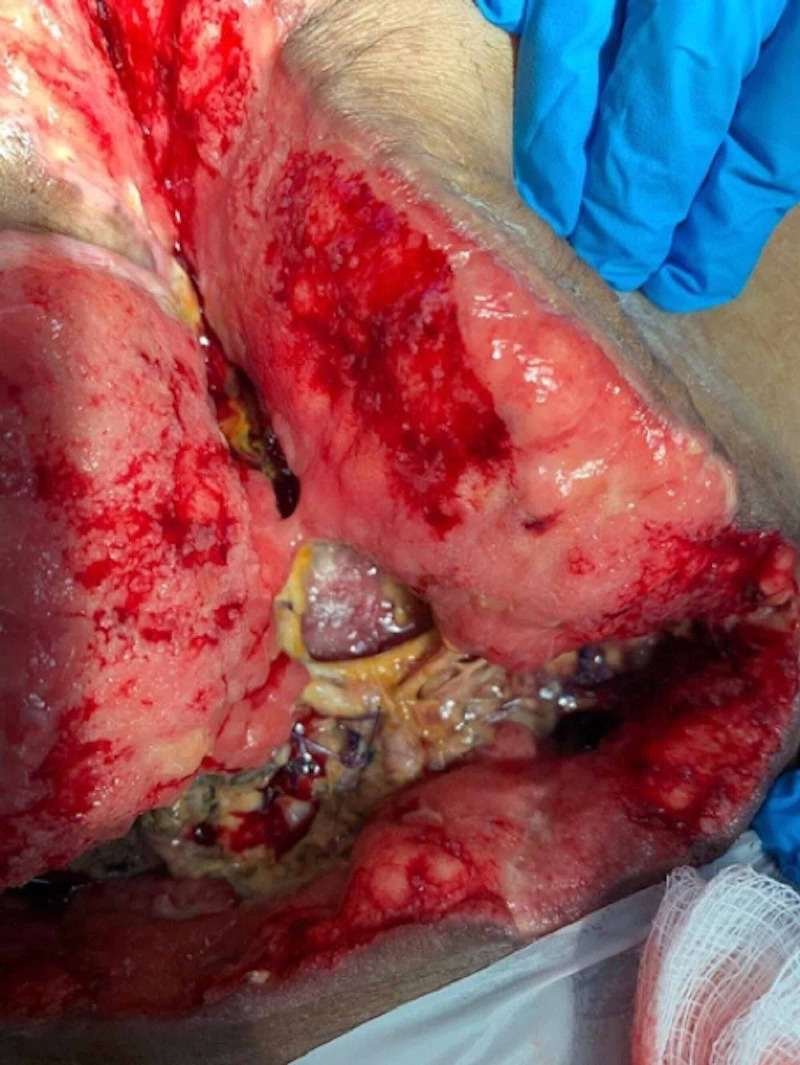
Entero-atmospheric fistula (EAF) on the left edge of the open wound

This is a 48-year-old female, a known case of hypothyroidism, who was admitted through the clinic after presenting with an entero-atmospheric fistula post ovarian cyst drainage complicated by bowel injury, managed by exploratory laparotomy with bowel resection (Figure 1).

**Figure 2:**
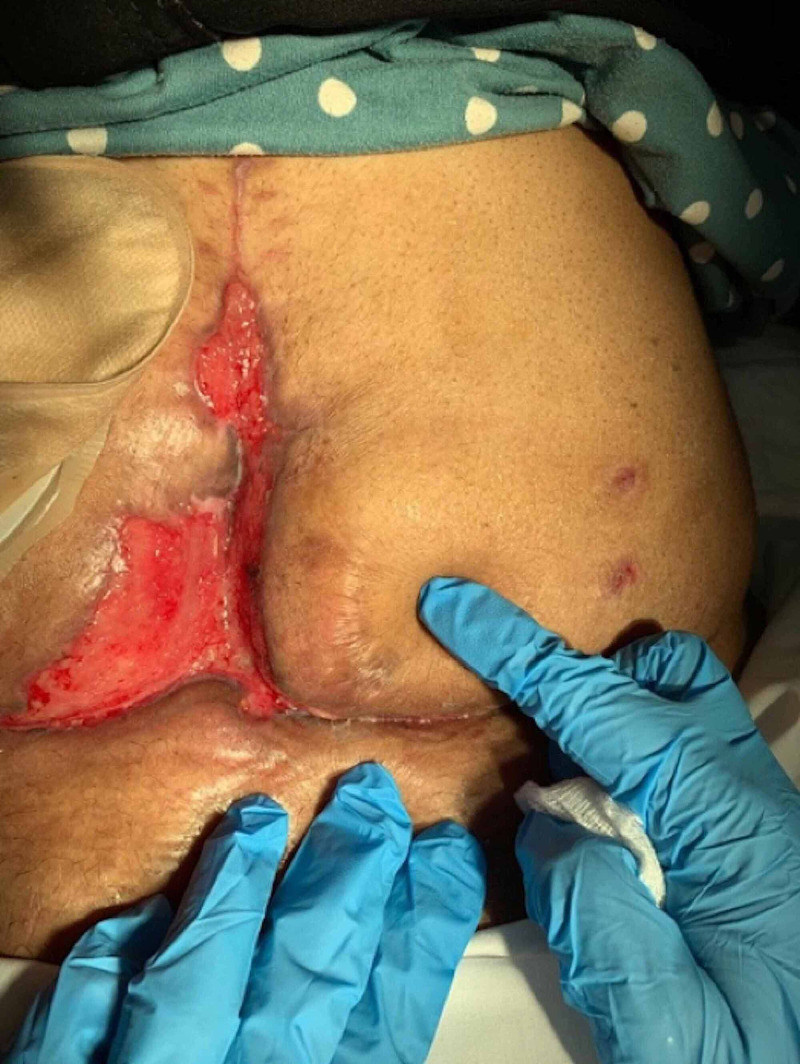
Wound during negative-pressure wound therapy

She has a history of three open surgeries (cholecystectomy, bowel resection, and ovarian drilling). She previously presented to the emergency room two times complaining of abdominal pain associated with fever, shortness of breath, and history of decreased appetite. She did not report nausea nor vomiting. She was passing stool and flatus. Physical examination showed guarding all over the peritoneum, and faecal matter was noted coming out of the surgical site. She was kept nothing by mouth "nil per os" (NPO) and a CT scan was arranged; intravenous fluid and intravenous antibiotics were started. Her vital signs were non-reassuring as her heart rate was 117 and her blood pressure was 78/64. She went to the operating theatre for exploratory laparotomy with a segmental small bowel resection and ileostomy with mucus fistula creation. Intra-operative findings were faecal contamination of the peritoneum, a perforated and ischemic ileum, necrotic fascia in the lower aspect of the abdomen and adhesions. Post-operatively, the plan was to use negative pressure wound therapy (NPWT), also known as vacuum-assisted closure (VAC), after ruling out foreign body existence, radiation exposure, distal obstruction, and sepsis (Figure 2).

**Figure 3:**
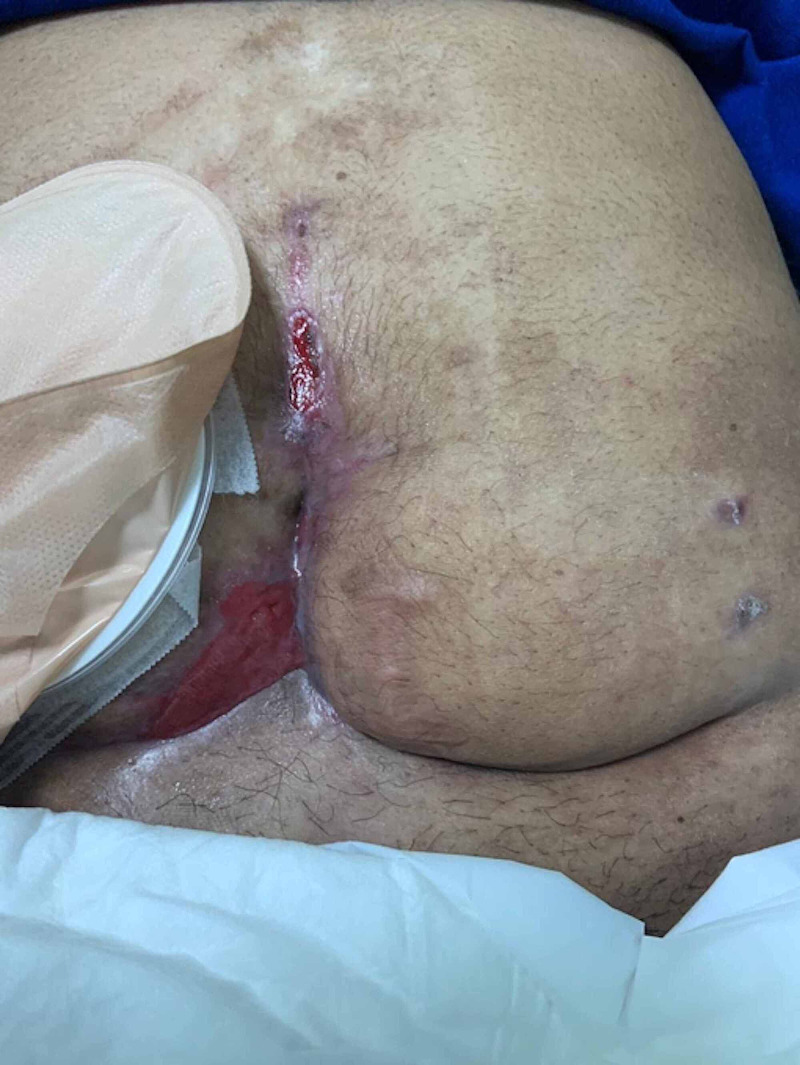
The wound after spontaneous fistula closure

We put a white sponge and a black one above it with intermediate pressure. VAC dressing output was 350 ml. In day 15 post-op, she underwent wound debridement because it had pus and an erythema around the wound and in the groin. She completed a course of tazocin for six days then switched to imipenem for nine days, then ciprofloxacin. Upon inspection wound was triangular in shape, ileostomy near the right side, there was purulent discharge and slough tissues at the wound base, and the fascia was intact. The plan was to discontinue VAC dressing, continue daily dressing, wound culture, and to be discharged home. Then she was admitted through the clinic as a case of entero-atmospheric fistula. We applied silver VAC GRANUFOAM™ (KCI, St. Paul, MN, USA) with 75 mm Hg pressure. A continuous therapy with medium intensity and dressing was done twice a week. A decrease in size, depth 95% closed with granulation tissue hyper-granulation. Now after three months the wound is completely healed (Figure 3).

## Discussion

EAF is defined as enteric fistula that is presented in an open abdomen. It occurs after trauma and any major abdominal procedures and is critical. Its management is complicated, leading to a higher cost. In addition, morbidity and mortality stay high despite contemporary medical care developments [1,2]. The treatment of EAF usually starts with a conservative approach, to avoid the hazards of major abdominal surgery. However, spontaneous healing is restrained by the absence of vascular tissue coverage over the exposed bowel [7]. To combat that, several techniques have been proposed such as the use of a catheter to intubate the fistula, early mobilization of skin and subcutaneous tissue, and the use of temporary absorbable mesh with selective vacuum-assisted wound closure [8,9]. In this case, the latter was used with the use of a VAC machine. The utilization and maintenance of the VAC machine were done according to the standardized methods, with a Jackson-Pratt drainage position over the fistula, and a separate suction tube applied over the drain with a negative pressure of 125 mmHg. Daily maintenance and change of VAC were done on a daily basis. Adjunct medical therapy consisted of prophylactic antibiotics along with total parenteral nutrition [10]. There have been several previous studies that investigated similar approaches. Tavusbay et al. investigated use of VAC for entero-atmospheric fistula and found four patients out of 33 to have spontaneous closure [11]. Similarly, Wirth et al. report a trial of three successful cases using a similar approach. However, a direct comparative study is needed to investigate the superior option between different modalities [12].

## Conclusions

In conclusion, EAFs are challenging to manage and require intensive monitoring and care due to an increased risk of morbidity and mortality. Although several techniques have been developed over the years and surgical intervention has been recognized as the mainstay of treatment of entero-atmospheric fistulas, the use of vacuum-assisted closure (VAC) to induce spontaneous healing can provide a safe acceptable alternative in selected patients. In this case, a 48-year-old female presented with an entero-atmospheric fistula post ovarian cyst drainage complicated by bowel injury, and VAC was used to aid in EAF healing which showed a satisfactory result.
